# Correction: Alkaline electrocatalytic water oxidation by Fe–Ni nanostructures on porous turbostratic carbon with tailorable metal–metal active sites

**DOI:** 10.1039/d6sc90011a

**Published:** 2026-01-15

**Authors:** Dipankar Saha, Chaoyun Tang, Javed Khan, Pulkit Jain, Cheng-Jie Yang, Chung-Li Dong, Richard F. Webster, Chi-Liang Chen, Zhu Chen, Peng Bai, Richard D. Tilley, Nianqiang Wu, James J. Watkins

**Affiliations:** a Conte Center for Polymer Research, Department of Polymer Science and Engineering, University of Massachusetts Amherst Massachusetts 01003 USA watkins@polysci.umass.edu; b Department of Chemical and Biomolecular Engineering, University of Massachusetts Amherst Massachusetts 01003 USA nianqiangwu@umass.edu; c Department of Physics, Tamkang University Tamsui 25137 Taiwan; d Mark Wainwright Analytical Centre, The University of New South Wales Sydney NSW 2052 Australia; e National Synchrotron Radiation Research Center Hsinchu 30076 Taiwan; f School of Chemistry, The University of New South Wales Sydney NSW 2052 Australia

## Abstract

Correction for ‘Alkaline electrocatalytic water oxidation by Fe–Ni nanostructures on porous turbostratic carbon with tailorable metal–metal active sites’ by Dipankar Saha *et al.*, *Chem. Sci.*, 2026, https://doi.org/10.1039/d5sc05330g.

The authors regret that the original article contained an incorrect spelling of the author name Richard D. Tilley. The correct author list is as shown here.

The Royal Society of Chemistry apologises for these errors and any consequent inconvenience to authors and readers.

